# The effect of jicama (*Pachyrhizus erosus L.*) starch on the properties and probiotic potential of *L. plantarum* SN13T fermented milk

**DOI:** 10.5455/javar.2024.k779

**Published:** 2024-06-08

**Authors:** Sri Melia, Indri Juliyarsi, Rizki D. Setiawan, Salam N. Aritonang, Hurriya Alzahrah, Doni Supandil

**Affiliations:** 1 Department of Animal Products Technology, Faculty of Animal Science, Universitas Andalas, Padang, Indonesia; 2 Department of Animal Production, Faculty of Animal Science, Universitas Andalas, Padang, Indonesia; 3 Student of Magister Program, Faculty of Animal Science, Universitas Andalas, Padang, Indonesia

**Keywords:** Cold storage, *Pachyrhizus erosus L.*, storage time, sensory evaluation, syneresis

## Abstract

**Objective:**

This study investigated the application of Jicama starch (*Pachyrhizus erosus L.*) as a stabilizing agent to enhance the longevity and integrity of fermented milk.

**Materials and Methods:**

*Lactobacillus plantarum* SN13T (6 gm/100 ml) and Jicama starch (2 gm/100 ml) were added into pasteurized milk (65°C, 30 min) and then incubated under anaerobic conditions at 37°C for 18 h. The fermented milk was stored at 4°C. The evaluation on proximate composition, pH, titratable acidity (TA), viscosity, water holding capacity (WHC), syneresis, total lactic acid bacteria (LAB), and hedonic sensory evaluation was conducted at 1, 7, 14, 21, and 28 days of storage.

**Results:**

Throughout the storage period, fermented milk enriched with Jicama starch significantly (*p* < 0.05) increased pH, TA, population dynamics of LAB, viscosity, WHC, and syneresis. It effectively sustained WHC and mitigated syneresis, thus ensuring the preservation of vital product quality. Furthermore, the quantity of LAB within the fermented milk consistently met the probiotic threshold of 84.50 × 10^8^ CFU/ml. The hedonic sensory evaluation results indicated that fermented milk showed consistent sensory attributes throughout storage, except for overall acceptance, which declined on day 28.

**Conclusion:**

The addition of Jicama starch revealed a promising health probiotic product, presenting a viable avenue for delivering probiotic benefits to consumers while maintaining the palatability and efficacy of the product.

## Introduction

The nutritional composition and potential health implications of food have recently garnered increased consumer attention. A noteworthy development in the realm of functional foods is the emergence of synbiotic foods, which synergistically combine probiotics and prebiotics, thereby offering various health benefits to the body [1]. Local tubers, including sweet potatoes and jicama, are rich sources of compounds such as dietary fibers, resistant starch, and fructooligosaccharides, which are efficient prebiotic components [2,3]. Prebiotics also play a role as thickening agents crucial for maintaining the high quality of yogurt by preventing syneresis, the unfavorable separation of liquid from the gel. Prebiotics derived from local tubers can gel, thereby increasing the viscosity required for yogurt production [4].

Jicama contains 5.8% moisture, 5.7% crude oil, 6.2% crude fiber, and 85% accessible carbs [5]. The starch derived from *Pachyrhizus* spp. is characterized by high viscosity, small particle size, and low pasting temperature [6]. Jicama extract has been reported to enhance the growth of *Lactobacillus *(*L.*)* plantarum* [7] and provides various health benefits, such as lowering blood sugar and enhancing immune response [3]. The use of hydrolyzed Jicama starch in yogurt does not significantly impact pH and viscosity but can affect the sensory properties of yogurt, with higher concentrations of jicama starch leading to sensory changes [8].

*Lactobacillus plantarum* is one of the commonly used lactic acid bacteria (LAB) serving as a starter culture in various food fermentations, imparting functional properties related to Echegaray et al. [9]. In our previous research, *L. plantarum SN13T* was isolated from stingless bee honey and exhibited potential as a probiotic bacterium [10]. However, the characteristics of synbiotic fermented milk products using *L. plantarum SN13T* as a probiotic and Jicama starch as a prebiotic remain unknown, especially regarding changes in characteristics during storage.

Therefore, this study aimed to examine the effects of Jicama starch on fermented milk during cold storage on physicochemical properties, LAB viability, and sensory qualities because fermented milk tends to lose thickness during storage due to syneresis or the expulsion of liquid from the gel. This study investigated the application of jicama starch (*Pachyrhizus erosus L.*) as a stabilizing agent to enhance the longevity and integrity of fermented milk.

## Materials and Methods

### Materials

Milk was obtained from livestock, the freeze-dried starter of *L. plantarum* SN13T was obtained from the Animal Products Technology Laboratory, and jicama starch was purchased from HASIL BUMIKU, Bantul, Jogya, Indonesia.

### Preparation of fermented milk

Fermented milk was made based on a modification of the method by Abdel-Hamid et al. [11]. Milk was pasteurized at 65°C for 30 min and then cooled until the temperature reached 28°C. After that, 6/100 ml starter and 2 gm/100 ml Jicama starch were inoculated, mixed evenly, and incubated under anaerobic conditions at 37°C for 18 h. It was then stored at a cold temperature of 4°C for 1, 7, 14, 21, and 28 days.

### Physicochemical analysis of L. plantarum SN13T fermented milk

The prepared fermented milk samples were analyzed for moisture, ash, protein, fat, and carbohydrate content [12]. The pH value of the samples was determined using an electrode pH meter (Hanna, Italy) and calibrated with pH four and pH seven standard buffers. 10 gm of yogurt was dissolved in 100 ml of distilled water [11]. To measure titratable acidity (TA), 10 gm of the sample was quickly dissolved in 30 ml of distilled water and carefully blended. A few drops of the phenolphthalein indicator were added to the combined solution. It was titrated with a standard solution of 0.1 N sodium hydroxide until a light pink hue persisted for around 10–15 sec to ensure complete neutralization [13].

Total TA = (ml NaOH × 0.009) × {weight of milk (gm)}-1 × 100%.

### Enumeration of LAB

1 g of each agitated sample was dissolved in 9 ml of buffer peptone water to create a serial dilution (to 10^–6^). LAB counts were calculated using MRS agar with pH 5.5 (neogen). The dishes were then incubated for 48 h at 37°C under anaerobic conditions. Colony-forming units per gram (CFU/gm) represent the results [14].

### Determination of viscosity

The viscosities of the samples were determined using a viscometer (Model NDJ-8S), according to the procedure modified by Akgun et al. [15]. The viscometer was operated at 1.5 and 3 rpm, and readings were recorded in centipoises. The average of the three measurements was determined.

## Determination of water holding capacity (WHC)

WHC was measured using a modified centrifugation method by Senaka et al. [16]. In summary, 5 g of samples were centrifuged at 1792 × g for 30 min. The centrifuge tube containing the sediment was weighed after removing the supernatant. The majority of the analyses were performed in triplicate. The WHC was calculated as follows:

WHC (%) = 1 – (W1/W2) × 100

where: W1 = Weight of whey after centrifugation, W2 = Fermented milk weight

### Determination of syneresis

The method Yapa et al. [14] measured syneresis to determine when it started to occur in samples while they were being stored in a refrigerator. 15 g of the sample were centrifuged at 640 × g for 20 min at 4°C using a tabletop centrifuge. The following formula is used to determine syneresis.


$$\mathrm{Syneresis}\;\mathrm{(\%)}=\frac{\mathrm{Volume}\;\mathrm{of}\;\mathrm{whey}\;\mathrm{separate}\;(\mathrm{ml})}{\mathrm{Sample}\;\mathrm{weight}\;(\mathrm{gm})}\;x\;100.$$

### Sensory analysis

The samples were evaluated by a panel of 25 individuals (13 women and 12 men, which is the age 18–23 years). Sensory characteristics of the product, such as aroma, taste, texture, color, and overall acceptability, were evaluated using a 1 to 5-point hedonic scale (5 = like extremely, 4 = like, 5 = neither like nor dislike, 4 = dislike, and 1 = dislike extremely). This method was a modification from [14].

### Statistical analysis

All observations were repeated three times. SEM was used to prepare the results. One-way analysis of variance was used to compare the means, followed by Duncan’s test (*p* < 0.05). SPSS version 26 was used for statistical analysis.

## Results and Discussion

### Physicochemical properties of fermented milk

Table 1 presents the physicochemical properties of *L. Plantarum* SN13T fermented milk without Jicama starch (control) and the difference in fermented milk with Jicama starch. In fermented milk containing Jicama starch, the moisture content decreased from 86.99% to 85.59%, lipid content decreased from 2.42% to 0.53%, and protein content decreased from 4.86% to 2.67%. The decrease in protein and fat after the addition of ijama starch to fermented milk is due to the carbohydrate content that has doubled compared to that without the addition of starch, which consequently suppresses the number of other substances such as protein and fat in fermented milk. In comparison, the ash content increased from 0.56% to 0.73%, and the carbohydrate content increased from 5.19% to 10.49%. This increase was caused by the high carbohydrate content of Jicama starch, which led to a significant increase in the total carbohydrate content and a reduction in the concentration of other nutrients in fermented milk like protein and fat. Jicama flour (*P. erosus L.*) contains 5.8% moisture, 5.7% crude fat, 6.2% natural fiber, 85% available carbohydrates, and 22.29% starch [5].

### pH

Changes in the pH of fermented milk were monitored for 28 days in this study (Table 2). The pH of *L. plantarum* SN13T fermented milk with the addition of jicama starch decreased significantly (*p* < 0.05) after 7 days of storage (4.3) and increased substantially (5.2) after 14 to 28 days of storage. In the first week, the availability of sufficient nutrients and the presence of Jicama starch increased the activity of *L. plantarum* SN13T, resulting in the increased activity of LAB in producing their metabolites. Starch is a source of energy for microbes to grow. However, on day 14th, the pH increased and the LAB grew significantly. This might be the peak of LAB growth thus the pH started to increase while the LAB remained high.

High bacterial metabolism converts lactose into lactic acid, acetaldehyde, diacetyl, and formic acid, which combine to lower yogurt pH. Naibaho et al. [17] explained that pH changes can occur during the fermentation process in yogurt supplemented with Brewers’ spent grain (BSG). BSG may contribute to the availability of amino acids for LAB growth. This effect may be due to the potential of BSG as a prebiotic due to its high dietary fiber and protein content. so that the increased growth of LAB increased the amount of acid which caused a decrease in pH.

Alcântara et al. [18] reported similar results for acai yogurt, where an increase in pH was observed at 21 and 28 days of storage following a significant decrease in pH values during the first 14 days of storage. LAB enter the stationary phase when they run out of nutrients or when toxic metabolic by-products accumulate during growth [19]. This study’s findings are nearly identical to those of Susmiati et al. [20], who reported that fermented milk with the addition of orange had a pH range of 3.57 to 4.23. Furthermore, adding carrot juice to fermented milk results in a pH of 4.48 to 4.53 [21].

### TA

Changes in TA of fermented milk after the addition of 1/100 gm Jicama starch during 28 days of storage were statistically significant *(p* < 0.05) (Table 2). TA of fermented milk decreased after 7 days of storage but increased again after 21 days of storage. This is also related to the growth of LAB that decreased during storage, resulting in a decrease in the amount of organic acids produced by LAB as their primary metabolites, which indirectly affects the TA value. The volume of organic acid produced by LAB influences the variation in the acidity of fermented milk [22].

This result was similar to that reported by Ehsani et al. [23], after 21 days of cold storage, symbiotic buffalo yogurt containing *Lactobacillus acidophilus* and *Bifidobacterium bifidum* had post-acidification quantities ranging from 4.53 to 3.80. This increase in acidity was a result of post-acidification. During the 28-day storage period, fermented goat milk produced by *Pediococcus acidilactici* BK01 had TA values ranging from 1.52% to 1.73% [24].

### Total LAB

Lactic acid is the primary flavoring component the starting bacteria produces during yogurt manufacturing and storage. Table 2 displays the changes in the number of LAB during 28 days of storage. The quantity of *L. plantarum* SN13T found in fermented milk with Jicama starch added ranged from 42.75 to 108 × 10^8^ CFU/ml. At 14 days of storage, LAB in fermented dairy grew significantly (*p* < 0.05) but dropped after 21 to 28 days. This is because the growth of *L. plantarum* SN13T was excellent for up to 14 days of storage, utilizing nutrients to the greatest extent possible. In the bacterial growth phase, bacteria increase their growth by maximally utilizing existing nutrients, but after reaching the stationary phase, nutrients are insufficient, causing a decrease in the growth of LAB during storage. In this regard, during the fermentation process and storage, LAB utilize ijama starch as their energy source.

According to Yapa et al. [14], the addition of bael extract affects the growth of *L. rhamnosus* GG during storage. After 7 and 14 days, probiotic yogurt containing bael extract was more significant than the control. Prebiotic elements in the bael extract, specifically fibers, are responsible for the increased number of bacteria. Shah et al. [25] state that nutrients, inhibitory agents, number of colonies supplied, incubation temperature, fermentation period, and storage temperature also influence the survivability of bacteria in fermented milk.

### Viscosity

The viscosity of *L. plantarum* SN13T fermented milk containing Jicama starch decreased significantly (*p* < 0.05) during storage (Table 2). Viscosity changes during the fermentation process as a consequence affect the textural formation, due to the fermentation. If the viscosity declines, the texture becomes less compact. The decrease in viscosity during storage is related to the reduction in total LAB count. As a result, the amount of primary metabolites, namely organic acids produced, also decreases, leading to an increase in pH. This change in pH affects the syneresis of the product, causing it to decrease along with the viscosity.

The fermentation process is essential for the growth of LAB as well as for the formation of gel formation in yogurt. The formation of gel formation affects the viscosity and consistency of yogurt during storage. The availability of protein in fermented yogurt has an impact on the formation of structures in yogurt, thus modifying the physical properties of yogurt. Casein micelles interacted with polysaccharides throughout the fermentation process, resulting in gelation as a result of complexation and interfacial stabilization. According to Naibaho et al. [26], yogurt supplemented with BSG, was able to maintain the consistency of yogurt during storage, as indicated by a stable level of syneresis. It also increases the ability of LAB to grow and survive during cold storage.

LAB form the gel in yogurt through acidification of casein micelle networks, and the strength of this gel, dependent on casein-casein bonds, is easily compromised by mechanical treatment due to lactic acid destabilizing casein, consequently impacting water binding, syneresis, texture, and viscosity during storage and leading to alterations in yogurt’s physicochemical properties [27].

### WHC

**Figure 1.**
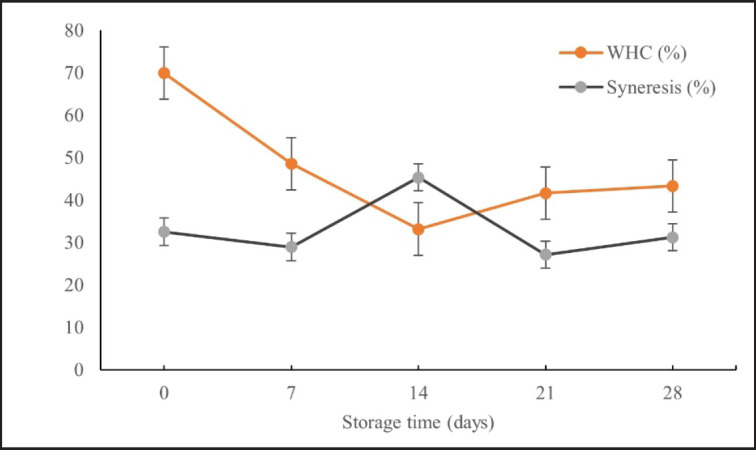
WHC and syneresis of fermented milk *L. plantarum *SN13T supplemented with jicama starch during storage.

Figure 1 shows the changes in WHC of *L. plantarum* SN13T fermented milk after 28 days of storage. On day 14, the WHC of fermented milk decreased significantly *(p* < 0.05), but increased after storage on days 21 and 28. This decrease in WHC was linked to the large number of LAB on day 14, which grows organic acids. Both the protein complex and micelle bonds are affected by acidity.

**Table 1.** Nutritional composition of fermented milk *L. plantarum *SN13T.

Nutrition	Fermented milk (control)	Fermented milk (jicama starch 2%)
Moisture (%)	86.99 ± 0.010	85.59 ± 0.424
Ash (%)	0.56 ± 0.007	0.73 ± 0.028
Fat (%)	2.42 ± 0.078	0.53 ± 0.035
Protein (%)	4.86 ± 0.099	2.67 ± 0.021
Carbohydrate (%)	5.19 ± 0.170	10.48 ± 0.014

**Table 2.** Physicochemical properties of fermented milk *L. plantarum *SN13T during storage.

Storage time (days)	pH	TA (%)	LAB (10^8^ CFU/ml)	Viscosity (Cp)
0	4.60 ± 0.41^b^	1.51 ± 0/21^a^	42.75 ± 14.82^b^	14264.88 ± 0.66^a^
7	4.30 ± 0.14^a^	1.26 ± 010^b^	47.25 ± 10.15^b^	4990.63 ± 0.89^b^
14	4.73 ± 0.09^b^	1.15 ± 0.07^b^	108.75 ± 3.46^a^	8332.50 ± 0.95^ab^
21	4.65 ± 0.06^b^	1.33 ± 0.10^ab^	57.00 ± 15,68^b^	6035.13 ± 0,54^b^
28	5.20 ± 0.18^c^	1.37 ± 0.12^ab^	84.50 ± 42,35^ab^	8438.36 ± 0,40^ab^

^a,b^The difference in superscripts in the same column indicates a significant difference (*p* < 0.05).

After 21 days, Jicama starch added to *L. plantarum* SN13T fermented milk can preserve WHC. This is influenced by the WHC of Jicama flour (*P. erosus* (L.) *Urban*), which was 363.88% [5]. According to Amatyakul et al. [28], adding stabilizers (starch, gum, pectin, and gelatin) can improve the consistency of yogurt, affecting its viscosity, syneresis, and WHC.

### Syneresis

Syneresis, or whey separation, is one of the determining factors affecting the quality and consumer acceptability of fermented milk. The addition of Jicama starch altered the syneresis of *L. plantarum* SN13T fermented milk during storage, as shown in Figure 1. Syneresis increased significantly *(p* < 0.05) on day 14 and decreased on days 21 and 28. This was consistent with the decrease in WHC in fermented milk observed in this investigation on day 14. Nonetheless, after 21 days of storage, both syneresis and WHC decreased.

**Figure 2.**
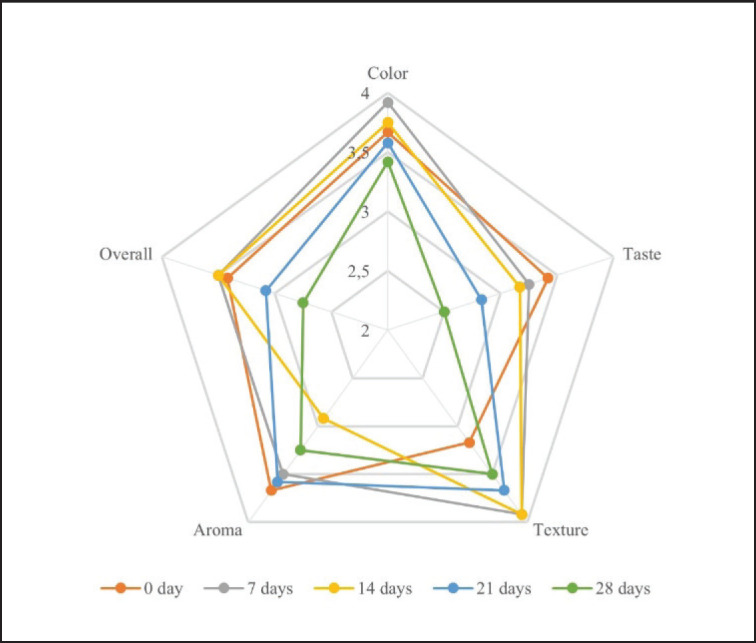
The sensory evaluation of fermented milk with *L. plantarum *SN13T supplemented with jicama starch during storage.

This starch can reduce syneresis. This is due to the stabilizing effect of Jicama starch, which is added to fermented milk as a stabilizer. Stabilizers have been introduced to products to regulate their texture and decrease whey separation [28]. Adding starch can increase the density of the gel network structure formation because starch promotes the construction of a compact network structure. In addition, Akgun et al. [15] noted that this change could have resulted from the formation of casein-micelles in the gel matrix and the rate of calcium solubility during storage.

### Sensory evaluation

The addition of Jicama starch in the milk fermentation process using *L. plantarum* SN13T did not significantly affect sensory attributes, except for overall acceptance, with color, texture, and aroma showing a non-significant increase during 7 and 14 days of storage followed by a decline after 28 days, while taste preference continuously decreased throughout the storage period, leading to significantly (*p* < 0.05) lower overall acceptance scores after 28 days (Fig. 2).

Despite the potential for changes in taste and texture during prolonged storage of fermented milk, attributed to the growth of undesirable microflora and microbial exopolysaccharides [29], this study found no significant differences in panelists’ acceptance of taste and texture over time. Additionally, the influence of prebiotic addition on color, aroma, texture, and overall customer acceptance depends on the specific characteristics of the prebiotic used, as noted by Gomes et al. [30].

## Conclusion

The research indicates that the addition of Jicama starch to fermented milk brings about significant changes in essential features during storage, including alterations in pH, TA, LAB population dynamics, viscosity, WHC, and syneresis. Notably, the impact of Jicama starch becomes pronounced after 28 days, effectively preserving water-holding capacity and reducing syneresis, ensuring the maintenance of crucial product quality. The quantity of LAB consistently exceeds the probiotic threshold, and up to 28 days of storage does not significantly affect panelists’ preferences for color, taste, texture, and aroma, except for an observed reduction in overall acceptance on the 28th day. This study highlights the potential of the product as a healthy probiotic option, offering a practical means of delivering probiotic benefits to consumers while maintaining product palatability and efficacy.

## List of Abbreviations

CFU, coloni forming unit; gm, gram; *L. Plantarum, Lactobacillus plantarum*; ml, mililiter; MRS, de Man, Rogosa, and Sharp; RPM, revolution per minute; SEM, Scanning Electron Microscope, TTA, titratable acidity; WHC, water holding capacity.
